# Gaze-cueing effect depends on facial expression of emotion in 9- to 12-month-old infants

**DOI:** 10.3389/fpsyg.2015.00122

**Published:** 2015-02-10

**Authors:** Alicja Niedźwiecka, Przemysław Tomalski

**Affiliations:** Neurocognitive Development Lab, Faculty of Psychology, University of WarsawWarsaw, Poland

**Keywords:** infant, gaze cueing, emotion expression processing, shared signal hypothesis, negativity bias

## Abstract

Efficient processing of gaze direction and facial expression of emotion is crucial for early social and emotional development. Toward the end of the first year of life infants begin to pay more attention to negative expressions, but it remains unclear to what extent emotion expression is processed jointly with gaze direction at this age. This study sought to establish the interactions of gaze direction and emotion expression in visual orienting in 9- to 12-month-olds. In particular, we tested whether these interactions can be explained by the negativity bias hypothesis and the shared signal hypothesis. We measured saccadic latencies in response to peripheral targets in a gaze-cueing paradigm with happy, angry, and fearful female faces. In the Pilot Experiment three gaze directions were used (direct, congruent with target location, incongruent with target location). In the Main Experiment we sought to replicate the results of the Pilot experiment using a simpler design without the direct gaze condition. In both experiments we found a robust gaze-cueing effect for happy faces, i.e., facilitation of orienting toward the target in the gaze-cued location, compared with the gaze-incongruent location. We found more rapid orienting to targets cued by happy relative to angry and fearful faces. We did not find any gaze-cueing effect for angry or fearful faces. These results are not consistent with the shared signal hypothesis. While our results show differential processing of positive and negative emotions, they do not support a general negativity bias. On the contrary, they indicate that toward the age of 12 months infants show a positivity bias in gaze-cueing tasks.

## Introduction

A number of studies have demonstrated infants' sensitivity to salient social cues, such as gaze direction and facial expressions of emotion from the first months of life (for a review see e.g., Frischen et al., 2007). The first year of life is a period of rapid changes in the processing of social information. While newborns preferentially orient to faces and face-like patterns (Johnson et al., 1991; Farroni et al., 2005), throughout subsequent months infants begin to learn to regulate their attention (Colombo, 2001; Wass et al., 2011) and gradually learn to share their attention between people and objects (Butterworth, 2004; Gredebäck et al., 2010). Despite great progress of research in this area, only modest literature exists on the question of how infants integrate multiple dynamic and multimodal social cues into meaningful entities. In our study we aimed to examine the effects of facial emotion and gaze direction on visual orienting toward the end of the first year of life. In subsequent sections we first review research on the effects of gaze direction on infant attention, followed by work on similar effects of emotion expressions. Finally, we outline potential mechanisms that explain the interactions between perceived emotion and gaze.

A number of authors demonstrated that visual orienting to gaze-cued targets is faster than orienting to uncued targets both in infants and in adults (for a review see Frischen et al., 2007). This effect was shown in many studies using a gaze cueing paradigm, itself based on a classical Posner spatial cueing paradigm (Posner, 1980). In the gaze cueing paradigm (Driver et al., 1999) a central cue (a face) is followed by a peripheral target. In one condition the gaze direction of the face predicts the target location, in the other condition the gaze direction cues the location on the opposite side of target location. The gaze-cueing effect is the facilitation of saccades made to the gaze-cued location relative to the saccades made to the non-cued target. The difference between saccadic latencies in these two conditions reflects the size of the effect.

The gaze-cueing effect has been demonstrated in 4 month-old infants by Farroni et al. (2000). Apart from the fact that orienting to gaze-cued locations was faster in comparison with uncued locations, the authors also showed that the perceived movement of the eyes is an important contributor to the effect (an illusion of gaze shift, see also Hood et al., 1998). Consistent with these findings was a study by Hoehl et al. (2014), who compared the effects of a gaze shift without a head turn and of a head turn without a gaze shift on infant orienting. Both a gaze shift and a head turn caused increased attention to the target. This study evidenced the importance of perceived movement (of the eyes or of the head) for the following of another's line of sight. Moreover, another person's gaze not only directs attention toward the gaze-cued object but also leads to enhanced processing of that object (Reid et al., 2004).

Direct gaze has a unique effect on human attention. Perceived eye contact modulates the processing of concurrent and immediately subsequent stimuli (Senju and Johnson, 2009; Johnson et al., in press). It is detected and preferred (over averted gaze) already at birth (Farroni et al., 2002). Four-month-olds show a novelty preference for faces with direct gaze and recognize better those faces that were first presented with direct gaze (Farroni et al., 2007a). At that age the neural processing of a face with direct gaze is enhanced in comparison with a face with averted gaze (Grossmann et al., 2007a). Direct gaze may be unique because it is an indication of another's attention to the infant (Reddy, 2003) and because it may signal a communicative intent. Infants follow actor's gaze only when the gaze shift is preceded by an ostensive cue like direct gaze or infant-directed speech (Senju and Csibra, 2008).

Toward the end of the first year of life, infants differentiate facial expressions of basic emotions (Hoehl et al., 2008; Kobiella et al., 2008; Martin et al., 2008). In 3-month-olds, the processing of a happy face, but not of a simultaneously presented object, is enhanced in comparison with a neutral face (Hoehl and Striano, 2010). Moreover, at the same age a fearful face enhances attention to objects cued by the face in comparison with a neutral one (Hoehl et al., 2008).

There is evidence to suggest that during the first months of life, infants prefer happy faces over other expressions. Newborns prefer smiling faces over fearful ones (Farroni et al., 2007b). At 4 months of age, infants still look longer at a happy face and orient toward it more often in comparison with an angry face. However, at 6–7 months of age, a developmental shift may occur, marked by increased attention to fearful faces. Six-month-olds, unlike 3-month-olds, show differential neural responses to fearful vs. neutral faces (Hoehl and Striano, 2010). Fearful facial expressions engage 7-month-olds' attention more than other expressions, as evidenced by longer looking times, larger Nc (a component of ERP associated with the orienting of attention to visual stimuli, Richards et al., 2010) and less frequent looks at distractors in comparison with happy expressions (Peltola et al., 2008, 2009, 2011). This effect of fearful face on infant attention may be an early indicator of developmental emergence of the negativity bias, i.e., a negative inclination in stimulus processing.

In adults the negativity bias has been shown to affect cognition at different stages of information processing, from rapid and automatic responses, such as orienting to images (Ito et al., 1998), through evaluation of stimuli and action preparation (Huang and Luo, 2006) to complex, explicit appraisals, such as political views (Hibbing et al., 2014). While there is some evidence for the presence of the negativity bias in older infants (for a review see Vaish et al., 2008), little is known about the effects of emotion expressions on information processing in the first year of life. Moreover, the dichotomy between positive and negative emotions alone is insufficient to explain the effects of gaze cueing by emoting faces on visual orienting.

Naturally occurring social stimuli are usually complex. A face conveys both the information on gaze direction and on the expression of emotion. The mechanisms underlying the combined effects of perceived gaze and emotion are not well understood. A theory proposed by Adams and Kleck (2003), states that humans process gaze direction and facial expressions of emotion more efficiently when they share a congruent (consistent) signal value. These authors demonstrated that gaze direction either enhances or hinders the processing of facial emotion expressions, depending on the behavioral tendency associated with a given emotion. In particular, direct gaze enhances the processing of approach emotions (anger and happiness), while averted gaze enhances the processing of avoidance emotions (fear and sadness) (Adams and Kleck, 2005).

To date, only a few studies tested this hypothesis in young infants. In an ERP study, 4-month-old infants showed an adult-like pattern of responses to faces conveying happiness and fear with direct vs. averted gaze, although the interactions between gaze and emotion were less pronounced than in adult participants (Rigato et al., 2010). In another study, direct gaze was associated with enhanced neural processing of angry expressions in 4 month-olds (Striano et al., 2006). These studies strongly suggest that there is an interaction between perceived gaze and emotion already in infancy. However, Matsunaka and Hiraki's (2014) findings from a behavioral task are inconsistent with that conclusion. They used static pictures of fearful and neutral faces to measure visual orienting in a gaze-cueing paradigm. Twelve-month-olds oriented faster toward targets cued by the fearful face compared with the neutral one. Six-months-olds did not show any facilitation of saccades by fearful faces. Concurrently, they did not find any effect of gaze direction (congruent vs. incongruent with target location vs. direct) on visual orienting or any significant interaction of gaze direction and emotion expression. These results are inconsistent with previous studies on gaze cueing (Farroni et al., 2000) and with studies on the eye contact effect (Senju and Johnson, 2009). One potential explanation for these discrepancies lies with the use of static stimuli by Matsunaka and Hiraki (2014), in particular, by the lack of movement of the eyes. As noted earlier, previous studies of gaze-cueing in infancy showed that gaze movement cues are important for eliciting the gaze-cueing effect (Hood et al., 1998; Farroni et al., 2000). We note that one way of dealing with this problem even when using static pictures is to adjust the presentation time of face stimuli with different gaze direction (e.g., from direct to averted) to produce an illusion of motion (perceived as a gaze shift).

Given the caveats revealed by existing work, we conducted two experiments to test the effects of emotion and gaze direction on visual orienting in infancy. Existing work provides very limited evidence for interactions of gaze and emotion under 12 months of age. Rigato et al. (2010, 2013) found robust interactions in adults and only limited interactions in infants. De Groote et al. (2007) also did not find strong evidence for this interaction in a gaze following task in 3, 6, and 9-month-olds. Therefore, while young infants distinguish facial expression of basic emotions and are sensitive to gaze direction, it seems that the interaction between emotional valence and gaze direction emerges later in development. For this reason, we first tested whether positive vs. negative emotions have differential effect on orienting in infants by comparing happy with angry and fearful faces. Furthermore, we also sought to examine whether two negative emotions are processed differently on the basis of their behavioral tendency (approach vs. avoidance). Rigato et al. (2013) compared fearful and sad face, which are both approach-oriented. We directly compared one approach-oriented (anger) and one avoidance-oriented (fear). We used a gaze-cueing paradigm to compare how these emotion expressions affect visual orienting in infants aged 9–12 months. For angry and happy faces, the behavioral tendency (approach) was inconsistent with gaze direction (away), while for the fearful face, the behavioral tendency (avoidance) was consistent with gaze direction (away).

Regarding our hypotheses, we first tested whether there is a gaze-cueing effect for emoting faces, predicting that orienting to gaze-cued targets is faster than orienting to uncued targets (Hypothesis 1). Secondly, we expected that the effect of emotion expression on visual orienting can be explained by the negativity bias, so that orienting to targets cued by negative faces (angry, fearful) is faster in comparison with a positive face (happy) (Hypothesis 2). Finally, we hypothesized that the differences in the processing of the two negative emotions can be explained by the shared signal theory (Hypothesis 3). Thus for the two negative expressions, we predicted faster orienting if behavioral tendency associated with the emotion is consistent with averted gaze (fearful face), than if behavioral tendency is inconsistent with averted gaze (angry face). See also Table 1 for the summary of predictions.

**Table 1:** **Predicted differences between saccadic reaction times (SRTs) in pairs of conditions (Emotion × Gaze direction)**.

**Hypothesis**	**Pairs of conditions**	**Rationale**
1	Angry congruent < Angry incongruent	Gaze-cueing effect
	Happy congruent < Happy incongruent	
	Fearful congruent < Fearful incongruent	
2	Angry congruent < Happy congruent	Negativity bias
	Fearful congruent < Happy congruent	
3	Fearful congruent < Angry congruent	Shared signal
		(congruent signal value for fear)

## Pilot experiment

In this pilot study we compared directly 3 expressions of emotion: angry, happy and fearful and 3 gaze directions: direct (straight ahead), gaze-congruent and gaze-incongruent. In order to increase the likelihood of infants looking at the eyes of emotional faces, at the beginning of each trial we first presented a neutral face gazing straight ahead, which served as an ostensive cue. We did not use a neutral face as a control condition, since young infants do not necessarily discriminate it from a happy face (Martin et al., 2008). This neutral face with direct gaze was then followed by one of three emotion expressions, which was either looking straight ahead (1/3 of trials) or sideways (remaining 2/3 of trials). The direct gaze condition served as a point of reference to compare with the effect of gaze cueing (in the gaze-congruent condition). On the trials with gaze shift the infants watched emoting faces gazing first straight ahead and then shifting the gaze sideways, thus the face immediately preceding the appearance of the target was gazing sideways.

### Method

#### Participants

Twenty-eight healthy infants between the ages of 8.8–12.2 months took part in the study. Fourteen participants were excluded from the analysis because they did not have at least one valid trial per condition and at least 12 valid trials in total. An additional infant was excluded due to extremely slow saccades (2 SD above the group mean). The final sample consisted of 13 infants (5 girls, mean age *M* = 9.9, *SD* = 1.2) who completed on average 16.1 valid trials (*SD* = 1.6; 89% of all trials).

Participants were middle-class families from a city with >1.5 million inhabitants. Mean maternal education was 17.2 completed years (*SD* = 1.03). The study was approved by the local institution's ethnics committee. All parents gave written informed consent prior to the testing.

#### Stimuli

Female faces with four different expressions of emotion (happy, angry, fearful, and neutral) were taken from the standardized NimStim set with the author's permission (Tottenham et al., 2009). Faces were presented centrally, subtending 14.04° (length) × 7.59° (width) of visual angle. One face identity was used for all four expressions. Photos representing frontal-view faces gazing forwards were modified to produce images gazing to either side: the gaze-relevant contrast was enhanced and irises and pupils moved to the either side, consistent with previous studies (Rigato et al., 2010). Targets were colorful pictures of toys, presented laterally, subtending 7.59° (length) × 7.59° (width) visual angle. The distance from face edge to the inner edge of the target was 7.59° visual angle at the viewing distance of 60 cm.

#### Procedure

The experiment took place in a purpose-built testing room. Infants were seated in a high chair or, if necessary, on parent's lap, approximately 60 cm from the stimulus monitor. Eye-tracking data was collected using a Tobii T60XL eye-tracker (Tobii Inc.) with a 24″ monitor, 60 Hz sampling rate and 0.5° accuracy (value provided by the manufacturer). A five-point infant-friendly calibration was performed, with each infant successfully calibrating at least 4 points. The entire task did not exceed 5 min.

**Figure 1:**
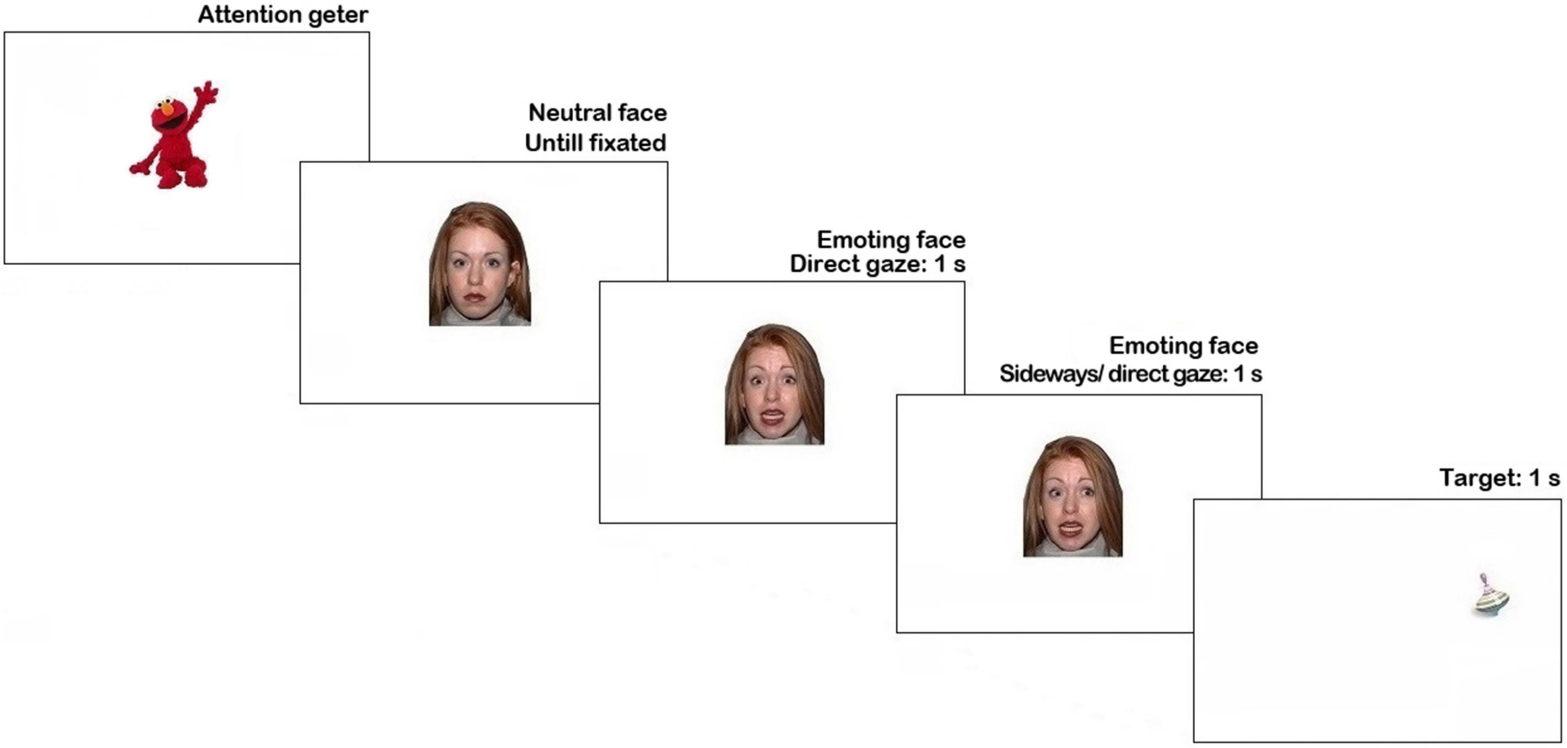
**Trial structure in Pilot experiment. N.B**. The face stimulus in the figure is different from the study set at the request of NimStim authors (see Tottenham et al., 2009).

The stimuli were presented using Tobii Studio 3.2 (Tobii Inc.). Trial sequence is represented in Figure 1. Each trial began with an attention getter (a spinning cartoon with sound) presented in the center of the screen until the infant fixated it or for a maximum of 2 s.

The attention getter was followed by a picture of a neutral face with direct gaze presented for 1 s, replaced by an emoting face with the same identity for another 1 s. Then, on 66% of trials, the emoting face shifted gaze either to the left or to the right side (each 33% of all trials), on the remaining 33% of trials it continued to display direct gaze. As soon as the face stimulus cleared off the screen, a visual target appeared for 1 s. on one side of the screen (sides were counterbalanced). On half of the trials with the gaze shift the target's location was congruent with the gaze direction, while on another half it was in the opposite location. The inter-trial interval was 1 s-long. Altogether each participant was presented with 18 trials (2 trials per condition), presented in a fixed pseudorandom order.

#### Eye-tracking data analysis

The Tobii Fixation Filter^1^ was used to smooth the eye-tracking data. The data were analyzed according to Areas-of-Interest (AOIs) drawn around the faces and the targets. AOIs for faces were ovals, with width and height equal to face's measurements. AOIs for targets were drawn around the pictures with a margin overall subtending 9.0° (length) × 9.0° (width) visual angle. For a trial to be considered valid, the infant had to look at the screen while the face and the target were being presented. Saccadic reaction times (SRTs) were calculated on the basis of the position of infant's eyes, as latency from the target onset to the first fixation on the target.

#### Statistical analysis plan

The SRT data were submitted into a 3 × 3 repeated-measures ANOVA with two within-subject factors: gaze direction (direct, congruent with target, incongruent with target) and emotion expression (happy, angry, and fearful). Where necessary, the Greenhouse-Geisser correction was used.

To further test whether orienting to gaze-cued targets is faster than to non-cued targets (Hypothesis 1) we conducted paired-samples *t*-tests for the following conditions: angry congruent vs. angry incongruent; angry congruent vs. angry direct; happy congruent vs. happy incongruent; happy congruent vs. happy direct; fearful congruent vs. fearful incongruent; fearful congruent vs. fearful direct.

The effects of facial expression and gaze direction on saccadic latencies were tested in the primary analysis of variance. Planned contrasts were used to clarify these effects in particular conditions. First, we tested Hypothesis 2, examining whether orienting to targets cued by negative expressions (angry, fearful) is faster in comparison with a positive expression (happy). We compared latencies for the angry congruent and the fearful congruent condition with the happy congruent condition.

Finally, for Hypothesis 3 we tested whether orienting to the target is faster when a negative emotion and gaze share a consistent behavioral tendency than if a negative emotion and gaze have inconsistent behavioral tendency. To this end planned contrasts between both negative expressions were conducted (fearful vs. angry congruent vs. incongruent; fearful vs. angry congruent vs. direct).

### Results and discussion

**Figure 2:**
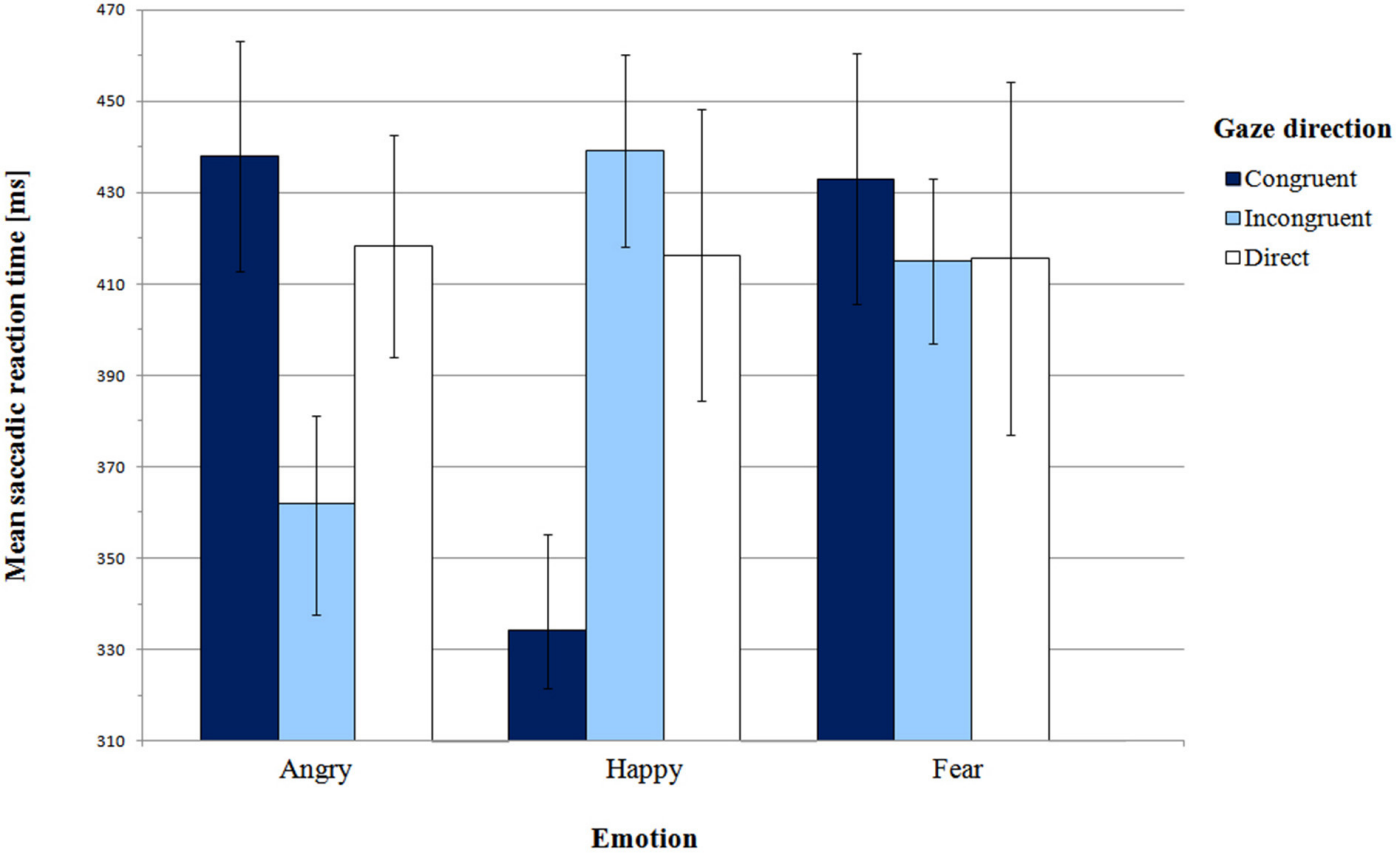
**Mean saccadic latencies in Pilot experiment**. Error bars represent standard error of mean.

Mean saccadic latencies in Pilot experiment with SEM for all conditions are presented in Figure 2 and supporting Table S1. The analysis of variance did not reveal any significant main effect of emotion [*F*_(2, 24)_ = 1.05, *p* = 0.36, η^2^_*p*_ = 0.08] or gaze direction [*F*_(2, 24)_ = 0.19, *p* = 0.83, η^2^_*p*_ = 0.02]. However, there was a highly significant interaction between these factors [*F*_(4, 48)_ = 6.97, *p* = 0.001, η^2^_*p*_ = 0.37].

First, we addressed Hypothesis 1, which stated that orienting to gaze-cued targets is faster than orienting to uncued targets. We did not find the gaze cueing effect for all emotion expressions. In order to examine the significant effect of interaction between gaze and emotion, we conducted pair-wise comparisons of congruent and incongruent gaze conditions for each emotion individually. For the happy face, SRTs in the congruent gaze condition were significantly shorter than in the incongruent condition [*M* = 334.15 ms vs. *M* = 439.19 ms, respectively; *t*_(12)_ = −5.25, *p* = 0.001, BCa 95% CI (−148.61, −61.47)]. Surprisingly, for the angry face there was a reverse pattern of results: latencies in the gaze-incongruent condition were significantly shorter than in the congruent one [*M* = 361.92 ms and *M* = 438.00 ms, respectively; *t*_(12)_ = 2.50, *p* = 0.03, BCa 95% CI (9.65, 142.50)]. For the fearful expression the latencies did not differ from each other [*t*_(12)_ = 0.50, *p* = 0.62, BCa 95% CI (−59.56, 95.33)].

Next, we compared SRTs between the gaze-congruent and the direct gaze condition for each emotion expression. We found a significant difference only for the happy expression, where latencies in the congruent condition were shorter than in the direct gaze condition [*M* = 334.15 ms and *M* = 416.35 ms, respectively; *t*_(12)_ = −2.56, *p* = 0.03, BCa 95% CI (−152.28, −12.10)]. There were no significant differences for the angry [*t*_(12)_ = 0.69, *p* = 0.51, BCa 95% CI (−42.87, 82.33)] or the fearful expression [*t*_(12)_ = 0.43, *p* = 0.67, BCa 95% CI (−70.18, 104.79)]. Altogether, we observed a gaze-cueing effect only for the happy expression, while for the angry face infants were slower to look at the cued than the non-cued target.

Hypothesis 2 concerned more rapid orienting to peripheral targets cued by negative than positive expressions. Although in the ANOVA we did not find a main effect of emotion, planned contrasts were used to compare congruent and incongruent conditions for happy vs. angry and fearful face. There was a significant difference between the happy expression and the two negative expressions in the congruent vs. incongruent condition [*F*_(1, 12)_ = 25.35, *p* = 0.001, η^2^_*p*_ = 0.68]. However, the results are different than we expected. In the congruent vs. incongruent gaze condition, shorter latencies were observed for the happy face in comparison with the two negative faces conditions. This pattern of results was confirmed by pair-wise comparisons of emotions in the gaze-congruent condition. SRTs in the angry face condition (*M* = 438.00 ms) were longer than in the happy face condition [*M* = 334.15 ms; *t*_(12)_ = 3.60, *p* = 0.01, BCa 95% CI (40.94, 166.76)]. In the fearful face condition (*M* = 433.00 ms), SRTs were also longer than in the happy face condition [*t*_(12)_ = 3.62, *p* = 0.01, BCa 95% CI (39.42, 158.27)]. Thus, Hypothesis 2 was not confirmed. Instead of shorter latencies for congruent negative expressions, we found significantly longer latencies for negative than happy faces.

In the direct gaze condition, saccadic latencies did not differ between expressions of emotion, as shown by paired comparisons [angry direct vs. happy direct: *t*_(12)_ = 0.08, *p* = 0.94, BCa 95% CI (−52.65, 56.49); angry direct vs. fear direct: *t*_(12)_ = 0.08, *p* = 0.94, BCa 95% CI (−64.44, 69.70); happy direct vs. fear direct: *t*_(12)_ = −0.03, *p* = 0.097, BCa 95% CI (−43.47, 42.16)].

Finally, according to Hypothesis 3, for the negative expressions we predicted shorter SRTs for the condition with consistent behavioral tendency (fearful face with averted gaze) compared with the condition with inconsistent tendency (angry face with averted gaze). Planned contrast for angry and fearful face (congruent vs. incongruent conditions) did not reveal any significant difference [*F*_(1, 12)_ = 1.56, *p* = 0.24, η^2^_*p*_ = 0.12]. SRTs in the fearful-congruent condition were not shorter than in the angry-congruent condition [one-sided paired samples *t*-test, *t*_(12)_ = 0.17, *p* = 0.44, BCa 95% CI (−60.78, 70.78)]. Moreover, in the congruent vs. direct condition, there was no significant interaction between fear vs. anger and gaze direction either [*F*_(1, 12)_ = 0.01, *p* = 0.95, η^2^_*p*_ = 0.01].

Taken together these results did not support our hypotheses, despite the evidence for differential processing of emotion expressions. Firstly, we found gaze-cueing effect only for the happy expression, with saccade facilitation to the gaze-cued side than to the non-cued side. Surprisingly, a reverse effect was present for the angry face, where saccades to the cued target location were significantly slower than saccades to the gaze-incongruent target. Furthermore, we did not find any difference in response times between the congruent and the incongruent or direct condition for the fearful face.

Given the existing literature on infant emotion processing we also expected a general bias toward negative expressions and toward objects cued by those expressions (Vaish et al., 2008). Also in this case the data did not support such a general attentional bias. Infants were faster to orient toward targets in the location cued by the happy face than by the fearful or angry face. Thus we have found the facilitation of visual orienting for the positive expression relative to both negative expressions.

Also a surprising result was the lack of emotion-related differences in the direct gaze condition. Infants showed highly similar saccadic latencies to peripheral targets preceded by a face with direct gaze, irrespective of the expression. It is possible that direct gaze overrides the effect of emotion. This is consistent with previous research showing that eye contact captures and holds infant attention (Senju and Johnson, 2009).

Consistent with the shared signal hypothesis (Adams and Kleck, 2003, 2005), we have further predicted the two negative expressions to differ in SRTs. However, our data did not give support to this hypothesis.

The results of this Pilot experiment may have somewhat limited significance because of two shortcomings. First, we note that some experimental effects may have been undetected due to a relatively small sample size of the study (participants included in the analysis, *n* = 13). The large number of participants excluded due to incomplete data may have affected the results, by potentially selecting those that are less fussy and more interested in the task. Secondly, we note that the inclusion of direct gaze trials in the design may have affected the overall results. On a third of trials the face did not appear to be shifting to either side, but it remained direct. Thus, we potentially had trials with stronger attention-holding effect than on trials with gaze shift occurring. To address these shortcomings a simplified paradigm without the direct gaze condition was used in the second experiment.

## Main experiment

In the second experiment we sought to replicate the results from the Pilot study in a larger sample. We simplified and modified the experimental design to control for several potential confounds. First, participants did not see a neutral face prior to being presented an emotion expression. In the Pilot study the neutral face was presented to ensure that infants attend to subsequent stimuli. However, attention getters displayed prior to each trial were sufficient in attracting infants' attention to the center of the screen. Second, we removed the direct gaze condition because we did not find any emotion-related differences in saccadic latencies in that condition. Moreover, by excluding this condition, we were able to increase the overall number of trials per condition without greatly increasing the duration of our task. Finally, stimuli with a second face identity were added to exclude the possibility that our results were specific to a single facial identity.

### Method

#### Stimuli

We used two female face identities from the NimStim set with fearful, happy, or angry expression. Otherwise, the stimuli were of the same size and position on the screen as in the Pilot experiment.

#### Procedure

**Figure 3:**
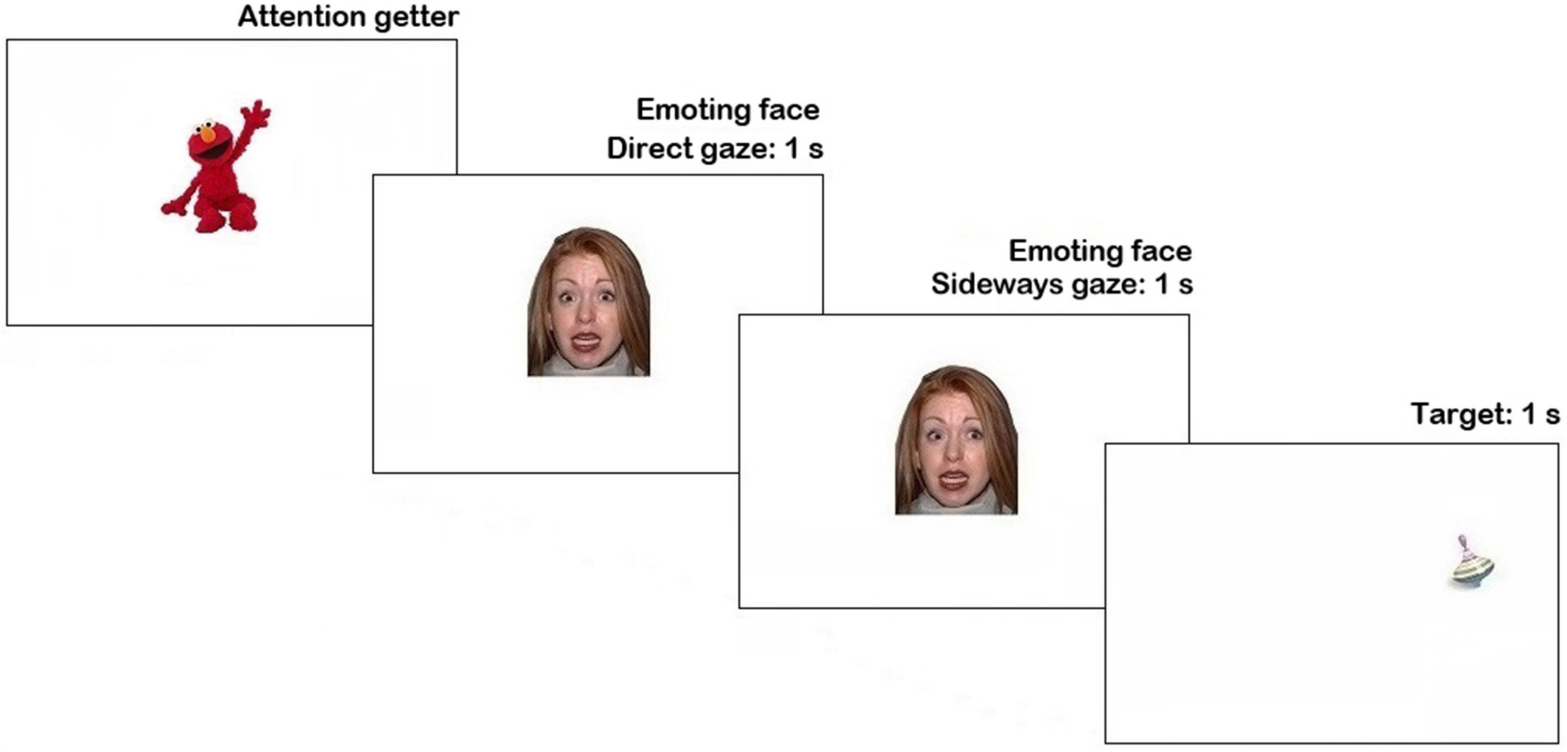
**Trial structure in Main experiment**.

The same testing procedures and eye-tracker software were used as in the previous experiment. The trial structure was modified to correct for potential confounds (see Figure 3). The attention getter was followed immediately by an emoting face expressing anger, happiness or fear with direct gaze (1 s duration), without any preceding neutral face as in Pilot experiment. Then, the face shifted gaze to the right or to the left (on 50% trials each), and remained on the screen for 1 s. As soon as the face stimulus was cleared off the screen a target picture of an attractive toy appeared for 1 s. On 50% of trials, the face looked in the direction, which predicted the location of a target, on another 50% of trials the target appeared on the opposite side of the screen. The inter-trial interval was 1 s-long. Altogether each participant was presented with 24 trials (4 trials per condition) in 2 blocks of 12 trials, with different face identity presented in each block, counterbalanced between participants. In Randomization 1, trials with face identity A were presented first, then trials with face identity B. In Randomization 2, trials with face identity B were presented first, followed by trials with face identity A. Equal number of participants were assigned to each randomization. To maintain participant's attention, a short clip from Sesame Street (35 s-long) was presented between the blocks. The duration of experiment did not exceed 5 min.

We tested the effects of stimulus order using a repeated-measures 3 × 2 × 2 ANOVA (Emotion × Gaze direction × Randomization) with randomization as a between-subjects factor. There was no main effect of stimulus order (randomization) on saccadic latencies [*F*_(1, 12)_ = 2.1, *p* = 0.17, η^2^_*p*_ = 0.15]. Moreover, there were no significant interactions between emotion and randomization [*F*_(2, 24)_ = 2.1, *p* = 0.15, η^2^_*p*_ = 0.16] or between gaze direction and randomization [*F*_(1, 12)_ = 1.88, *p* = 0.20, η^2^_*p*_ = 0.14]. Therefore, the results from two randomizations were analyzed altogether.

#### Participants

Thirty-eight healthy infants between the ages of 9.0–12.6 months participated in the study. Eight children were excluded from the analysis because they did not have at least 2 valid trials per condition and at least 12 valid trials in total. Next, three infants were excluded due to extremely long latencies (mean SRTs above 2 SD of the group mean). The final sample consisted of 27 infants (13 girls, mean age *M* = 10.6 months, *SD* = 1.1), who completed on average 18 valid trials (*SD* = 4.1; 75% of all presented trials).

Participants were recruited from the same area as in the Pilot experiment. Mean maternal education was 17.5 completed years (*SD* = 1.7). The study was approved by the local institution's ethnics committee. All parents gave written informed consent prior to the testing.

#### Eye tracking data analysis

The procedure of data analysis was exactly the same as in the Pilot experiment.

#### Statistical analysis plan

The SRT data were submitted into a 3 × 2 repeated-measures ANOVA with two within-subject factors: gaze direction (congruent with target, incongruent with target) and emotion expression (happy, angry, fearful). Where necessary, the Greenhouse-Geisser correction was used. To verify whether age is a significant factor, we re-run the analyses with age in weeks a covariate.

The presence of the gaze-cueing effect (Hypothesis 1) was further tested using paired samples *t*-tests for the following conditions: angry congruent vs. angry incongruent; happy congruent vs. happy incongruent; fearful congruent vs. fearful incongruent.

Next, we tested Hypothesis 2 whether orienting to targets cued by negative expressions (angry, fearful) is faster in comparison with a positive expression (happy). To this end we used planed contrasts, comparing latencies for the angry congruent and the fearful congruent condition with the happy congruent condition.

For Hypothesis 3, we tested whether orienting to the target is faster when the negative emotion and the gaze direction share a consistent behavioral tendency than if the emotion and gaze are associated with inconsistent behavioral tendency. To this end planned contrasts were run between both negative expressions (fearful vs. angry × congruent vs. incongruent).

### Results and discussion

**Figure 4:**
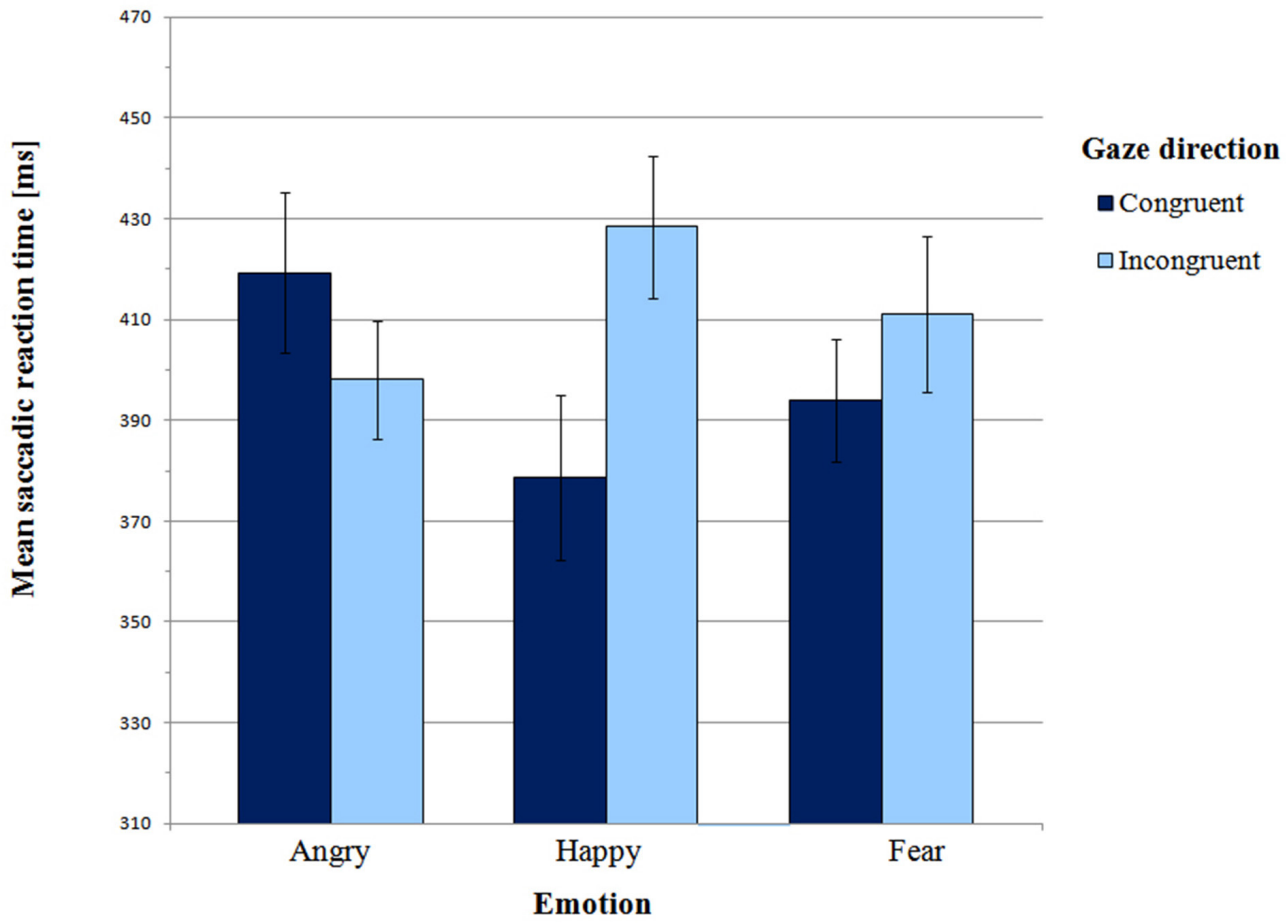
**Mean saccadic latencies in Main experiment**. Error bars represent standard error of mean.

Mean saccadic latencies in the Main experiment are presented in Figure 4 and supporting Table S2. A 3 × 2 (emotion × gaze direction) ANOVA revealed a significant interaction between emotion and gaze direction [*F*_(2, 52)_ = 5.32, *p* = 0.01, η^2^_*p*_ = 0.17]. There was no main effect of emotion [*F*_(2, 52)_ = 0.20, *p* = 0.92, η^2^_*p*_ = 0.01] or gaze direction [*F*_(1, 26)_ = 2.73, *p* = 0.11, η^2^_*p*_ = 0.10].

A 3 × 2 ANOVA with age (in weeks) as a covariate did not reveal any significant main effect of age [*F*_(1, 25)_ = 0.80, *p* = 0.39, η^2^_*p*_ = 0.03]. There weren't any significant interactions between age and emotion [*F*_(2, 50)_ = 0.68, *p* = 0.93, η^2^_*p*_ = 0.003] or age and gaze direction [*F*_(1, 25)_ = 0.009, *p* = 0.93, η^2^_*p*_ = 0.001] or three-way interaction between age, emotion, and gaze direction [*F*_(2, 50)_ = 0.95, *p* = 0.40, η^2^_*p*_ = 0.04]. Moreover, age did not correlate with SRTs in any of the conditions (all *p*s > 0.3). Therefore, participant age was not included in subsequent analyses.

Paired-samples *t*-tests were carried out to further test the presence of the gaze-cueing effect in individual emotions. For the happy face, mean SRT in the congruent condition was significantly shorter than in the incongruent condition (*M* = 378.58 ms vs. *M* = 428.38 ms, respectively; *t*_(26)_ = −3.37, *p* = 0.002, BCa 95% CI (−80.17, −19.43)]. Thus, there was a gaze-cueing effect for the happy face. However there were no differences between the congruent and the incongruent condition for both angry faces [*t*_(26)_ = 1.22, *p* = 0.23, BCa 95% CI (−14.50, −56.99)] and fearful faces [*t*_(26)_ = −1.19, *p* = 0.25, BCa 95% CI (−46.90, 12.59)].

According to Hypothesis 2, orienting to targets cued by a positive expression should be slower than orienting to targets cued by negative expressions. Planned contrast indicated a significant difference between the happy expression and the two negative expressions in the congruent vs. incongruent condition [*F*_(1, 26)_ = 7.10, *p* = 0.01, η^2^_*p*_ = 0.22]. This effect was driven by differential effect of angry vs. happy expression on gaze direction [*F*_(1, 26)_ = 11.13, *p* = 0.003, η^2^_*p*_ = 0.30]. As in the Pilot experiment, for gaze-cued targets SRTs were significantly shorter in the happy face condition than in the angry face condition (*M* = 378.58 ms vs. *M* = 419.27 ms, respectively; *t*_(26)_ = −2.34, *p* = 0.03, BCa 95% CI (4.90, -76.49)]. However, in the gaze-incongruent condition, SRTs were shorter for angry than for happy faces [*t*_(26)_ = −2.47, *p* = 0.02, BCa 95% CI (−55.64, −5.07); M angry incongruent = 398.02 ms vs. M happy incongruent = 428.38 ms]. Planned contrast between happy and fearful expressions did not show any significant interaction with gaze direction [*F*_(1, 26)_ = 2.02, *p* = 0.17, η^2^_*p*_ = 0.07]. In the gaze-congruent condition, saccadic latencies did not differ between the happy and the fearful face condition [*t*_(26)_ = −1.0, *p* = 0.33, BCa 95% CI (−47.09, 16.31)].

Finally, we tested Hypothesis 3, i.e., whether for negative expressions orienting is faster if emotion and gaze share a consistent signal value. Planned contrast between angry and fearful expressions in the congruent vs. incongruent condition was approaching significance [*F*_(1, 26)_ = 3.3, *p* = 0.08, η^2^_*p*_ = 0.11]. A one-tailed paired-samples *t*-test also revealed a trend [*t*_(26)_ = 1.60, *p* = 0.06, BCa 95% CI (−7.27, 57.88)], indicating that SRTs were shorter in the fearful congruent than in the angry congruent condition (*M* = 393.97 ms vs. *M* = 419.27 ms, respectively). Therefore, anger and fear may be processed differently, and these differences seem consistent with the shared signal hypothesis.

In conclusion, our data from the Main experiment are consistent with the data we obtained in the Pilot experiment. In particular, the gaze-cueing effect was only observed for the happy face. For the fearful face, saccadic latencies did not differ between the gaze-congruent and the gaze-incongruent condition. However, for the angry face, SRTs also did not differ between the gaze-congruent and gaze-incongruent conditions, unlike in Pilot experiment, where we found a significant difference (longer SRT's in the gaze-congruent condition).

Our second hypothesis also was not confirmed. Visual orienting was not faster for targets cued by negative expressions. On the contrary: orienting was faster for the happy face. This result was similar to the result in the Pilot experiment.

Notably, in the Main experiment we did find a difference approaching significance in orienting in the angry vs. fearful conditions. This result suggests that two negative emotions: one approach-oriented (anger) and the other one avoidance-oriented (fear) may be processed differently, as stated by the shared signal hypothesis (Adams and Kleck, 2003, 2005).

## General discussion

In our study we investigated the effects of emotion expression and gaze direction on overt orienting of 9- to 12-month-olds. Using a gaze-cueing paradigm we tested the effects of emotion expressions on the latency of saccadic responses to peripheral targets that were either cued by the eyes of the emoting face (congruent gaze condition) or on the opposite site of the cued location (incongruent condition).

There are two main findings from the initial Pilot experiment that were replicated in a simplified paradigm in the Main experiment. While we did not find overall faster orienting to gaze-cued targets than to uncued targets (Hypothesis 1), we did find a robust gaze-cueing effect the positive facial expression only. Infants oriented more rapidly to peripheral targets appearing in the location cued by the direction of the eye-gaze of happy faces compared with targets that appeared in the uncued location. Second, we found faster orienting to gaze-cued targets that were preceded by happy faces than to targets preceded by either angry or fearful faces. These results suggest that facial expressions of emotion affect the processing of gaze direction in infants younger than 12 months of life. We also found strong evidence for differential processing of positive and negative emotion expressions in the gaze-cueing task.

To our knowledge this is the first study to directly compare within-subjects the effects of a positive expression (happy) and two different negative expressions (angry fearful) on gaze cueing in infants at the end of the first year of life. Matsunaka and Hiraki (2014) compared only fearful and neutral expressions in a gaze-cueing paradigm. They did not find a gaze-cueing effect for either expression. However, they found a main effect of emotion, indicating that saccadic latencies were faster for targets cued by fearful face. With regards to the lack of gaze-cueing effect for the fearful expression our results are consistent with Matsunaka and Hiraki's (2014). However, we did not observe an overall more rapid orienting for fearful relative to happy or angry faces (no main effect of emotion found). Overall, the results from these two studies are difficult to compare due to differences in the experimental paradigm. It possible that Matsunaka and Hiraki (2014) found no gaze-cueing effect even for the neutral face because of the lack of perceived gaze shift. In the absence of a gaze shift (perceived movement of the eyes), the infants may have had insufficient gaze cues. The emotional cue was more salient than the gaze cue, hence they found only a main effect of emotion. In our experiments, we provided a perceivable gaze shift by presenting briefly a face with direct gaze prior to a face with averted gaze. Perhaps for this reason we were able to observe a gaze-cueing effect in at least one facial expression (happy). Further research is needed to explain the lack of gaze-cueing for angry and fearful face in our data.

The presence of the gaze-cueing effect for the happy face is in line with a computational model of the development of gaze following, according to which infants learn that interesting things appear where someone else is looking (Moore and Corkum, 1994; Triesch et al., 2006). Infants may look where someone else is looking because they notice the referential link between the person and the object. A positive expression could convey interest in an object. Infants are beginning to recognize such object-directed acts (e.g., looking at something) before their first birthdays (Carpenter et al., 1998; Brooks and Meltzoff, 2002, 2005; Woodward, 2003; Johnson et al., 2007). Finally, as episodes of positive affect are longer than episodes of negative affect in face-to-face interactions in typical, low-risk dyads (see e.g., Tronick, 1989), infants are most likely to perceive a smiling face during such interactions. Perhaps young infants learn to expect interesting things to appear where a smiling person is looking, as this is what they experience most often in naturalistic environment. Future research will show, if the perception of positive facial expression enhances infant learning in naturalistic settings.

One source of predictions for our experimental data came from research on attentional biases toward stimuli with negative emotional valence in childhood (Vaish et al., 2008). In the adult literature there is evidence for heightened neural responsivity to negative visual stimuli (Smith et al., 2003) and for enhanced neural responses to stimuli that appeared in the same location as fearful but not happy faces (Pourtois et al., 2004). Greater sensitivity and faster responses to negative than positive expressions in adults and children has been linked with their threat-relevance (LoBue, 2009). Consistent with this idea infants as young as 3 months show increased attention allocation and enhanced processing of objects cued by eye-gaze of faces with fearful relative to neutral expressions (Hoehl et al., 2008). Given these results, Hypothesis 2 predicted more rapid orienting to objects at a location gaze-cued by fearful and angry faces compared with happy expressions. Our data do not support the idea that negative emotion enhances gaze cueing. Orienting was faster in the happy face condition relative to the angry face condition (both experiments) and to the fearful face condition (Pilot experiment) but only in gaze-congruent trials. Therefore, happy face may enhance orienting in comparison with angry and fearful face but only as long as it is predictive of target location. It is possible that no advantage was found for negative expressions because they were more strongly holding infant attention prior to target onset, consistently with previous research (e.g., Peltola et al., 2008). Another explanation for finding gaze cueing effect for the positive condition only is that the negativity bias causes infants to be distracted by negative emotion (directing their attention away from the gaze cue).

There is some evidence that emotion expression and gaze direction are processed jointly. In particular, information processing may be enhanced if expression and gaze share a consistent signal value: approach or avoidance (Adams and Kleck, 2003). Drawing on the shared signal hypothesis (Adams and Kleck, 2005), we therefore predicted differential responses to fearful vs. angry expressions because of their opposite behavioral tendencies (avoidance vs. approach, respectively). In Hypothesis 3 we predicted more rapid orienting to peripheral targets when the behavioral tendency associated with the emotion is consistent with averted gaze (fearful face), than when the behavioral tendency is inconsistent with averted gaze (angry face). Our results did not offer clear support for this prediction. In the Pilot experiment we did not find a significant difference in the gaze-congruent condition for the angry vs. the fearful face. However, in the Main experiment, there was a difference approaching significance, consistent with the shared signal hypothesis. In particular, visual orienting was marginally faster when gaze and emotion shared a consistent signal value (averted gaze and fear), than when their signal value was inconsistent (averted gaze and anger). However, the results for the happy condition are inconsistent with this hypothesis.

Previous data indicates that the processing of negative emotion expression undergoes significant developmental changes between 6 and 12 months of age. Hoehl and Striano (2010) found a shift in neural responses to fearful vs. neutral faces between 6 and 9 months, while Grossmann et al. (2007b) showed similar change for happy vs. angry expressions between 7 and 12 months of age. Although we did not propose any specific hypothesis regarding the age-related change in our study, the relatively large age range of our sample, from 9 to 12 months, allowed us to test age effects in our data. While the Pilot study had insufficient sample size to conduct this analysis, in the Main experiment we did not find any effects of participant age on task responses. Thus despite the evidence in the literature that infant neural responses to different emotion expression change in the second part of the first year of life, this observation is not supported by behavioral data in our study. This is surprising given that the sequence of stimuli was similar to the paradigm used by e.g., Hoehl and Striano (2010). One possibility is that behavioral measures are less sensitive to age-related change than measures of cortical brain activity. Another possibility is that the development of emotion processing in the infants' brain is to a large extent latent throughout the period in question and its behavioral manifestations appear only in the coming months, as infants' gross motor development allows them to move more independently.

Taken together, our data may suggest the existence of a positivity rather than negativity bias in gaze cueing in the first year of life. In particular, happy face may facilitate visual orienting to other objects. Infants may not need the negativity bias to survive because they are taken care of and protected by their caregivers. From the point of view of the attachment theory (for a review, see Bretherton, 1993), pre-locomotor infants display fewer exploratory behaviors (moving away from caregiver) in comparison with attachment behaviors (staying close to caregiver). As a consequence, caregivers do not need to use negative expressions to keep infants away from danger. When the infants are capable of walking away from the caregiver, they need to use caregivers' referential gaze and facial expression to learn about danger in the environment. There is evidence from 13-month-olds that walking allows for better monitoring of caregiver's face than crawling (Kretch et al., 2014). Therefore, attentional bias to negative stimuli may be clearly observable in the second year of life (for a review, see Vaish et al., 2008). Infants younger than 12 months in our study were less independent in their exploration and to a larger extent reliant on their caregiver to detect danger in the environment. Further research is necessary to examine the relationship between locomotor skills and infant's sensitivity to caregiver's gaze.

While we were able to replicate the two main findings of our study, the results are subject to several limitations. In particular, there is a disproportion between positive and negative emotions in the design of the study. Participants saw twice as many negative as positive expressions, which may have influenced the processing of the stimuli, i.e., happy faces were less frequent, therefore more salient than negative faces. To provide a stronger test of the negativity or the positivity bias, an equal number of positive and negative stimuli should be displayed.

In summary, our results suggest that infant overt orienting between the ages of 9 and 12 months is influenced by interactions of gaze direction and facial expression of emotion. Across two experiments we found consistent gaze-cueing effect only for happy, but not for angry or fearful expressions. More rapid orienting to targets cued by happy faces relative to angry and fearful faces suggests the presence of a positivity bias in early infancy. While we found differential processing of approach- and aversion-oriented negative emotions, our results did not provide conclusive evidence for joint processing of gaze direction and negative emotion in this age group.

### Conflict of interest statement

The authors declare that the research was conducted in the absence of any commercial or financial relationships that could be construed as a potential conflict of interest.

## Footnotes

^1^The following settings were used: Eye selection: Average; Velocity Threshold: 35 pixels; Distance Threshold: 35 pixels; Interpolation for missing data for sections below: 100 ms. More information on the Tobii Fixation Filter can be found in the Tobii Studio 3.2 User Manual, available at manufacturers website: http://www.tobii.com/en/eye-tracking-research/global/library/manuals/
