# Supplementary material for: Gaze-cueing effect depends on facial expression of emotion in 9- to 12-month-old infants
Source: Front Psychol. 2015 Feb 10;6:122. doi: 10.3389/fpsyg.2015.00122 (PMC4322542; doi:10.3389/fpsyg.2015.00122)
Supplement: Supplementary file 1 [file Table1.DOCX]

*Table S1.* Mean saccadic reaction times in milliseconds in Pilot experiment (SEM in brackets).

| ***Gaze direction*** | ***Emotion*** | | |
| --- | --- | --- | --- |
|  | **Angry** | **Happy** | **Fear** |
| ***Congruent*** | 438.00 (25.18) | 334.15 (12.58) | 433.00 (27.49) |
| ***Incongruent*** | 361.92 (19.34) | 439.19 (20.93) | 415.16 (18.07) |
| ***Direct*** | 418.30 (23.40) | 416.35 (31.90) | 415.69 (38.56) |
