# Supplementary material for: Gaze-cueing effect depends on facial expression of emotion in 9- to 12-month-old infants
Source: Front Psychol. 2015 Feb 10;6:122. doi: 10.3389/fpsyg.2015.00122 (PMC4322542; doi:10.3389/fpsyg.2015.00122)
Supplement: Supplementary file 2 [file Table2.DOCX]

*Table S2.* Mean saccadic reaction times in milliseconds in the Main experiment (SEM in brackets).

| ***Gaze direction*** | ***Emotion*** | | |
| --- | --- | --- | --- |
|  | **Angry** | **Happy** | **Fear** |
| ***Congruent*** | 419.27 (16.00) | 378.58 (16.32) | 393.97 (12.20) |
| ***Incongruent*** | 398.02 (11.70) | 428.38 (14.08) | 411.12 (15.37) |
